# Glossiness Index of Objects in Halftone Color Images Based on Structure and Appearance Distortion

**DOI:** 10.3390/jimaging8030059

**Published:** 2022-02-27

**Authors:** Donghui Li, Midori Tanaka, Takahiko Horiuchi

**Affiliations:** 1 Graduate School of Science and Engineering, Chiba University, Yayoi-cho 1-33, Inage-ku, Chiba 263-8522, Japan; horiuchi@faculty.chiba-u.jp; 2 Graduate School of Global and Transdisciplinary Studies, Chiba University, Yayoi-cho 1-33, Inage-ku, Chiba 263-8522, Japan; midori@chiba-u.jp

**Keywords:** glossiness assessment, structure similarity, appearance distortion

## Abstract

This paper proposes an objective glossiness index for objects in halftone color images. In the proposed index, we consider the characteristics of the human visual system (HVS) and associate the image’s structure distortion and statistical information. According to the difference in the number of strategies adopted by the HVS in judging the difference between images, it is divided into single and multi-strategy modeling. In this study, we advocate multiple strategies to determine glossy or non-glossy quality. We assumed that HVS used different visual mechanisms to evaluate glossy and non-glossy objects. For non-glossy images, the image structure dominated, so the HVS tried to use structural information to judge distortion (a strategy based on structural distortion detection). For glossy images, the glossy appearance dominated; thus, the HVS tried to search for the glossiness difference (an appearance-based strategy). Herein, we present an index for glossiness assessment that attempts to explicitly model the structural dissimilarity and appearance distortion. We used the contrast sensitivity function to account for the mechanism of halftone images when viewed by the human eye. We estimated the structure distortion for the first strategy by using local luminance and contrast masking; meanwhile, local statistics changing in the spatial frequency components for skewness and standard deviation were used to estimate the appearance distortion for the second strategy. Experimental results showed that these two mixed-distortion measurement strategies performed well in consistency with the subjective ratings of glossiness in halftone color images.

## 1. Introduction

The halftoning technique is commonly used in the printing industry to reproduce the tone of an image with limited colors, e.g., black and white. The earliest error diffusion (ED) was proposed by Floyd and Steinberg [1,2]. However, early ED algorithms had problems with distortion, reduced visibility, worms and false textures, as well as additional noise. Later, references [3,4] improved Floyd’s ED. Pang et al. preserved the similarities of tone and structure in ED [5]. Akarun et al. improved the dithering halftone by using variable thresholds [6]. To overcome these shortcomings, Xia et al. developed two convolutional neural networks (CNNs) to learn the halftone scheme by using a nontrivial self-supervision formulation [7]. Thus, improvements to the ED algorithm were implemented with the goal of improving “image quality”. With the maturity of halftone technology, how to evaluate halftone accurately and objectively is still an unsolved problem. Most image glossiness assessment studies [8] are all device-based, i.e., based on photometer measurement. In the study of gloss perception in an image, it is currently debated whether the perception of gloss is linked to the statistical parameters of the retinal image [9,10,11,12]. Wiebel et al. analyzed many images of natural surfaces to search for potential statistical correlations of perceived gloss [13]. Pont and Koenderink found that skewness correlated with gloss when using rendered stimuli; however, the standard deviation, a measure of contrast, correlated better with perceived gloss when using photographs of natural surfaces [14]. Finally, the study verified the key role of contrast by manipulating the skewness and contrast within images. The structural similarity (SSIM) index [15] has become a standard in the image processing field. Studies in Refs. [16,17,18] used multiple strategies for image quality assessment (IQA). However, although the proposed methods simulated the human visual system (HVS) as much as possible, they could not be applied to the assessment of halftone images directly.

To evaluate the glossiness of objects in halftone images, the isolated dots in halftone images should be processed first. The dot process is a proposed method for material printing [19,20]. However, the method only searches for the inter-dot relationship measurement, spectral characteristics between dots, and the image spatial characteristics. Lee et al. proposed an innovative halftone IQA approach for color images [21]. HVS characteristics were applied to the proposed method, which used color spatial HVS filters for luminance, red-green, and blue-yellow components. This model effectively measured color distortion consistent with that of a human observer. However, the index evaluated “image quality” and could not be used to evaluate the gloss of an object.

Here, we propose a fully referenced image glossiness index for halftone color images. Instead of treating image distortions in the same way, we assume that they are decoupled into two groups: structural dissimilarity (SDSIM) and appearance perception, which would be correlated in diverse ways with visual non-glossiness and glossiness perception.

## 2. Related Work

Given a distorted image, humans can easily rate image quality. To eliminate the shortcomings of evaluation due to human observers, many researchers have focused on the assessment of computational models based on HVS [22,23,24]. The image quality metrics developed for traditional images are usually meant for multi-level (8-bit) images; hence, they cannot be applied directly to 1- or 2-bit discrete images. To overcome this issue, it is necessary to devise a process (e.g., a visual filter) to increase gradation.

To the best of our knowledge, the earliest halftone assessment index was proposed by Näsänen [25]. Näsänen’s method used an exponential function for the luminance component to evaluate a dithering halftone image. Later, Lee et al. proposed a new exponential function for evaluating halftone color images [21]. To consider the visual color characteristics, they performed a new function for each luminance, red-green, and blue-yellow components. Then, the color SSIM was applied to the reference and distorted images for the purpose of localized structural difference assessment. The index in [21] is useful for non-gloss images because their approach is based on SSIM, so it is effective for non-gloss objects with rich structural information. If there is an object such as a mirror, for a single-strategy index, they work well on all parts of the glossy object that reflect the environment surrounding it. However, for gloss objects, the proposed methods would fail because the glossy part contains little structural information. Therefore, to realize the correct assessment of an image, which contains glossy and non-glossy parts objectively, a single assessment strategy could not meet this demand.

In contrast to single-strategy image assessment methods, studies in Refs. [16,17,18] used a variety of strategies to simulate HVS closely for image assessment. In [26], instead of treating the image distortions equally, they proposed treating distortions as linear frequency distortions and additive noise degradations. However, this algorithm only focused on the halftone artifacts. Furthermore, the methods proposed in [17] did not solve the problem of combining the separated distortion measurement. Based on [27], they proposed two simple quality measures, i.e., the detail loss measure and the additive impairment measure, and developed a method of adaptively merging the two strategies. In [16], it was assumed that the HVS determined the image quality by performing different strategies and modeled them together using trained parameters. To solve the texture resampling low tolerance problem for the image assessment index, Ding et al. designed a monotonous and differentiable function using a CNN. Through this function, the image was transformed into a multi-scale representation [18]. Then, IQA metric that mixed correlations of texture similarity and correlations of structure similarity was developed.

Inspired by these strategies, this study develops an image glossiness index for halftone color images based on structure and appearance distortion.

## 3. Proposed Method

**Figure 1:**
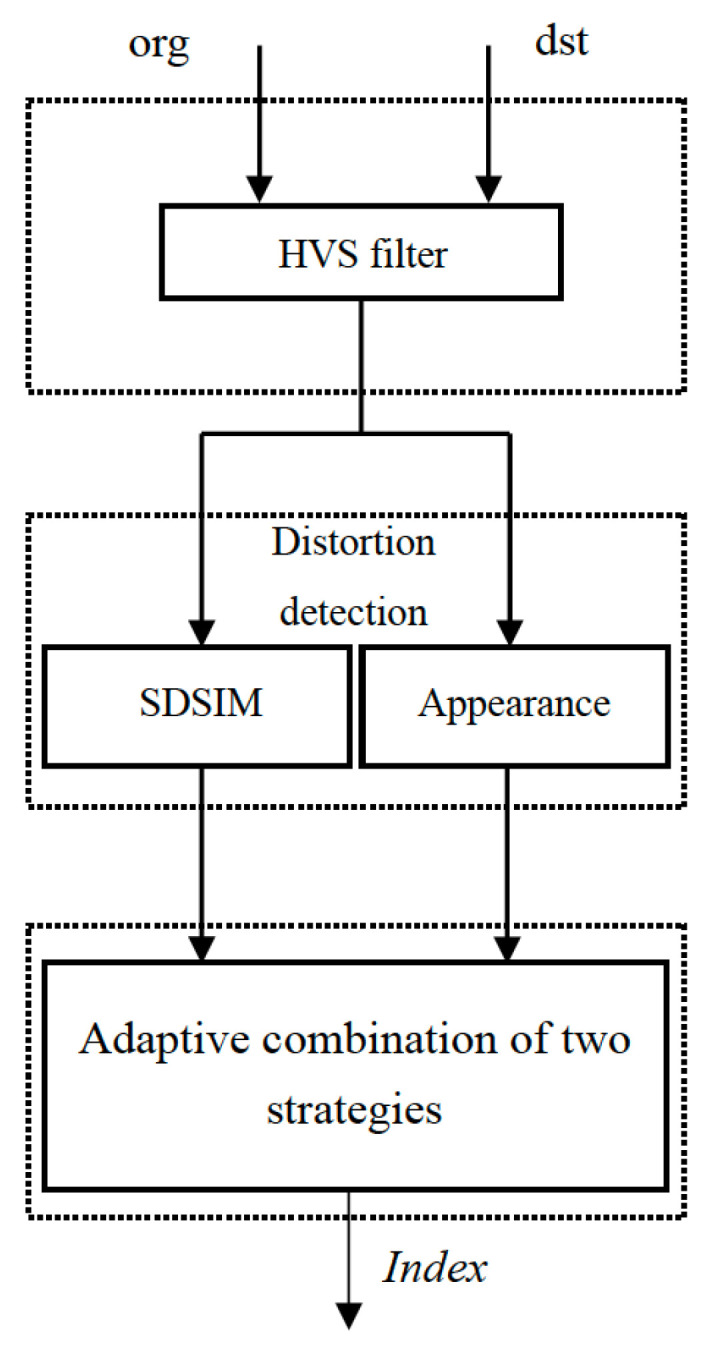
Flowchart of the proposed method.

The most apparent distortion (MAD) method was proposed by Larson [16]. In this paper, the author uses two strategies for distortion detection: the first strategy is based on the high-quality image detection strategy, which uses the mean squared error (MSE) to calculate the distortion of visibility. The MSE is the simplest and most widely used full-reference quality metric, which is computed by averaging the squared intensity differences of distorted and reference image pixels. The second detection strategy is based on appearance distortion. The strategy uses standard deviation, skewness, and kurtosis to calculate the distortion of low-quality images. Based on the idea of the MAD algorithm, the proposed algorithm (The execute file is available at https://github.com/donghuilee2022/IQA-for-halftone-image/tree/master, accessed on 12 February 2022) also evaluates the gloss of the image through two strategies. In the first strategy, we remove the HVS processing in the first strategy of MAD and use SDSIM replace MSE to calculate the distortion of visibility. In the second strategy, we only use the standard deviation and skewness related to gloss perception to calculate the appearance distortion. In addition, we also use a new HVS filter to preprocess the image. Finally, the adaptive method proposed in MAD is used to merge two strategies. The details are presented in the following subsections. First, we explain the near-threshold distortions using a method for quantifying perceived distortion, which was used to model the HVS detection. Second, we explain suprathreshold distortion, which was used to model HVS statistical characteristics. Third, we used a parametric method to combine the two modeled perceptual distortions, thereby achieving a single perception of overall distortion. Figure 1 presents a flowchart of the proposed algorithm.

### 3.1. HVS Filter

A visual perceptual model was used in our algorithm to consider the color characteristics of the HVS, which is described in Section 3.1.1 and Section 3.1.2.

#### 3.1.1. Color Space

The RGB images were transformed to CIEXYZ and then to CIELAB color space. In our experiments in Section 4, we assume color space is sRGB. XYZ is a special set of tristimulus values used for transforming between Lab and RGB. We use $L^{*}$, $a^{*}$, and $b^{*}$ to represent the components of CIELAB, respectively. $X_{n}$, $Y_{n}$, and $Z_{n}$ are tristimulus values with the D65 white point.

(1) $$L^{*}=\left\{\begin{array}{cc} 116\cdot {\left(\frac{Y}{Y_{n}}\right)}^{\frac{1}{3}}-16 & for\;Y/Y_{n}>0.008856\\ 903.3\cdot (\frac{Y}{Y_{n}}) & otherwise \end{array}\right.$$

(2) $$a^{*}=500\cdot \left(f\left(\frac{X}{X_{n}}\right)-f\left(\frac{Y}{Y_{n}}\right)\right)$$

(3) $$b^{*}=200\cdot \left(f\left(\frac{Y}{Y_{n}}\right)-f\left(\frac{Z}{Z_{n}}\right)\right)$$

(4) $$where\;f\left(t\right)=\left\{\begin{array}{cc} t^{\frac{1}{3}} & for\;t>0.008856\\ 7.787\cdot t+\frac{16}{116} & otherwise \end{array}\right.$$

#### 3.1.2. Contrast Sensitivity Function

To further improve the accuracy of the model to simulate the HVS, we chose the following exponential function for the luminance contrast sensitivity function.

(5) $$W_{\left(L^{*}\right)}\left(\tilde{\rho }\right)=K\left(L\right)e^{-\alpha \left(L\right)\tilde{\rho }}$$

(6) $$K\left(L\right)=aL^{b}$$

(7) $$\alpha \left(L\right)=\frac{1}{c\ln(L)+d}$$

The luminance for an image was represented by $L[cd/m^{2}]$, ρ (cycles/degree) was the spatial frequency, and *a* = 131.6, *b* = 0.3188, *c* = 0.525, *d* = 3.91. In contrast to Näsänen’s model, $\tilde{\rho }$ was defined as the weighted magnitude of ρ=(u,v):

(8) $$\rho =\sqrt{u^{2}+v^{2}}$$

(9) $$\phi =\arctan\left(\frac{v}{u}\right)$$

(10) $$s\left(\phi \right)=\frac{1-\omega }{2}\cos4\phi +\frac{1+\omega }{2}$$

(11) $$\tilde{\rho }=\frac{\rho }{s(\phi )}$$

The value of ω set to 0.7. s(ϕ) is a weight function. At odd multiples of $45^{\circ }$, this function reduced the contrast sensitivity to the components of the spatial frequency.

Comparing the sensitivity of human observers to spatial variations in luminance and to spatial variations in chromaticity, it was found that the latter decreased faster as the spatial frequency increased. The HVS chrominance model used here was based on Mullen’s [28]. The chromaticity CSF

(12) $$W_{\left(a^{*},b^{*}\right)}\left(\rho \right)=Ae^{-\alpha \rho }$$

The parameters α and *A* were set to 0.419 and 100 for the chrominance component.

**Figure 2:**
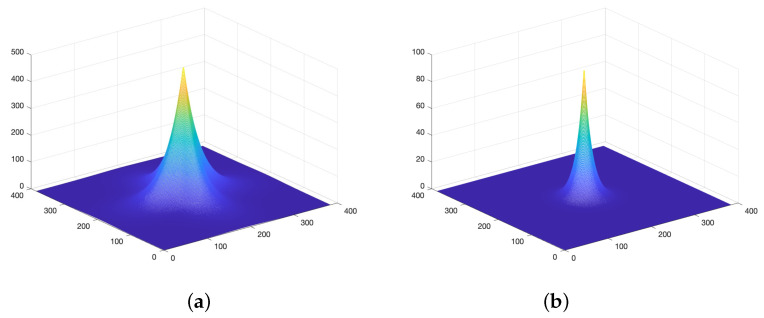
(**a**) Luminance frequency response model. (**b**) Chrominance frequency response model.

Using a chromaticity response model that was different from the above luminance resulted in low-frequency chromaticity errors, which were difficult to perceive by HVS. Figure 2 shows two different frequency response models, which represent the luminance and chromaticity response models.

In the luminance model, the weighting function effectively reduces the contrast sensitivity to spatial frequency components at odd multiples of $45^{\circ }$. In the chrominance model, the contrast sensitivity of the human observer to spatial variations in chrominance falls off faster as a function of increasing spatial frequency than the response to spatial variations in luminance.

### 3.2. Sdsim Distortion Detection Strategy

We used the visible distortion location calculation method proposed in MAD, but not to conduct the CSF filter. We only used perceived luminance and contrast masking methods in the high-quality assessment of MAD.

#### 3.2.1. Calculation of the Locations of Visible Distortion

Let $I_{org}$ and $I_{dst}$ represent the reference and halftone images, respectively, both of which were processed by the HVS filter in Section 3.1.

Perceived luminance: The reference and distorted images were transformed to luminance images via:

(13) $$L={\left(b+kI\right)}^{\gamma }$$

where *L* represents the luminance image and the parameters *b* = 0, *k* = 0.02874, and γ = 2.2. $L_{org}$ and $L_{dst}$ were calculated using the above equation. To consider the HVS nonlinear response to luminance, $L_{org}$ and $L_{dst}$ were transformed to luminance perception images ${\hat{L}}_{org}$ and ${\hat{L}}_{dst}$ via:

(14) $$\hat{L}=\sqrt[{3}]{L}$$

${\hat{L}}_{err}={\hat{L}}_{org}-{\hat{L}}_{dst}$ is defined as the error image.

Contrast masking: This masking explained the fact that image presence reduced the distortion detectability. First, the original image was divided into several 16 × 16 blocks; the change in the block size changed the average contrast of each block. The local contrast map corresponding to each block was calculated. Second, we calculated the root mean square (RMS) contrast for each block. The RMS contrast for block *p* of $I_{org}$ was calculated as:

(15) $$C_{org}\left(p\right)={\tilde{\sigma }}_{org}\left(p\right)/\mu_{org}\left(p\right)$$

where the mean value of block *p* is $\mu_{org}\left(p\right)$, and ${\tilde{\sigma }}_{org}\left(p\right)$ is calculated from the standard deviations of the four sub-blocks of *p*. $C_{org}\left(p\right)$ represented the local RMS contrast measurement of the original image, which was separated from the distortion of the image. Then, a local contrast map was calculated for the error image, which explained the distorted spatial distribution of the distorted image. In addition, $I_{err}$ was divided into 16 × 16 blocks, corresponding to the original image $I_{org}$. Each block’s RMS contrast $C_{err}\left(p\right)$ was calculated via:

(16) $$C_{err}\left(p\right)=\left\{\begin{array}{cc} \sigma_{err}\left(p\right)/\mu_{org}\left(p\right) & if\;\mu_{org}\left(p\right)>0.5\\ 0 & otherwise \end{array}\right.$$

where $\sigma_{org}\left(p\right)$ represents the standard of block *p* in $I_{err}$. The lightness threshold of 0.5, which explained why the HVS is insensitive to variations in dark regions. Finally, $C_{org}\left(p\right)$ and $C_{err}\left(p\right)$ are used to calculate the local distortion visibility map ξ(p):

(17) $$\xi (p)=\left\{\begin{array}{c} \ln C_{err}\left(p\right)-\ln C_{org}\left(p\right)\;if\;\ln C_{err}\left(p\right)>\ln C_{org}\left(p\right)>\delta \\ \ln C_{err}\left(p\right)-\delta \;if\;\ln C_{err}\left(p\right)>\delta >\ln C_{org}\left(p\right)\\ 0\;otherwise \end{array}\right.$$

specifically, ξ(p) reflected the amount by which the contrast of the error was larger than the contrast of the original image, if their contrast was greater than the threshold (δ=−5).

#### 3.2.2. The Combination of Local Structure Errors and Visibility Map

After calculating the visible location map, we use visibility-weighted local SDSIM which is used in the lightness domain to obtain the distortion of the perceived structure. The MSSIMMap is the MSSIM distribution map of one image, and SD(p) is calculated from the MSSIM mapping as follows:

(18) $$SD\left(p\right)=\frac{1}{16^{2}}\sum_{i,j\in N_{p}}{MSSIMMap}^{2}$$

where quantity SD(p) represents the local SDSIM calculated for each 16×16 block *p*. $N_{p}$ is the set of pixels inside block *p*, *i*, and *j* is the position of the pixel (i,j) in block *p*. MSSIM is the mean of SSIM, which was proposed in [18]:

(19) $$MSSIM\left(m,n\right)=\frac{1}{M}\sum_{j=1}^{M}SSIM\left(x_{j},y_{j}\right)$$

where *m* and *n* represent the original and halftone images. $x_{j}$ and $y_{j}$ are the image contents at the local window of *j*th, and *M* is the total number of local windows. Therefore, the perceived distortion $d_{sdsim}$ was computed by:

(20) $$d_{sdsim}={\left\{\frac{1}{P}\sum_{p}{\left[\xi (p)\times SD(p)\right]}^{2}\right\}}^{\frac{1}{2}}$$

**Figure 3:**
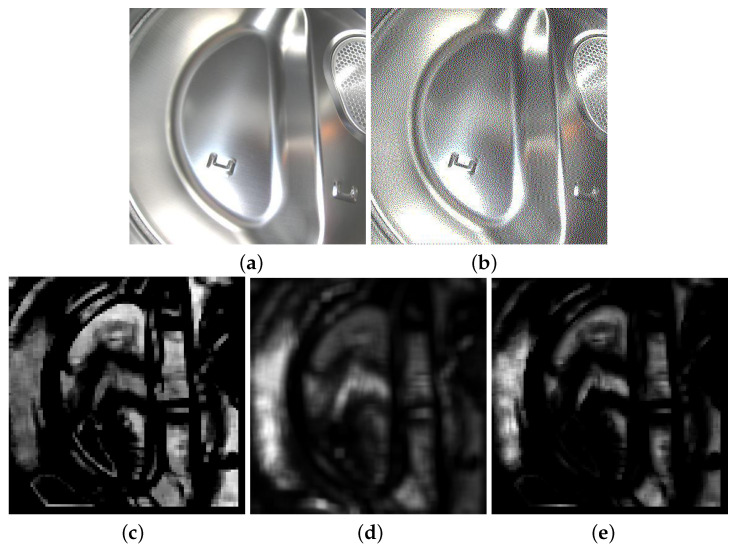
(**a**) Original image, (**b**) Floyd, (**c**) visible map, (**d**) SDSIM, and (**e**) perceived distortion.

Equation (20) represents a single value calculated from the visibility-weighted local SDSIM by using the L2 norm, which represented the sum of the visual structure of image distortion. $d_{sdsim}=0$ meant that the distortion in the distorted image would not be perceived by the visual system, i.e., it was not visible. The larger the value of $d_{sdsim}$, the greater the distortion perceived. Figure 3 shows the images of the maps involved in the $d_{sdsim}$ computation for a halftone image. Figure 3a,b show the original and halftone images, respectively.

These figures showed the calculated visibility map, local SDSIM map, and visibility-weighted local SDSIM map. In Figure 3c, the visibility map captured visible artifacts. In Figure 3d, the local SDSIM indicated that the greatest distortions appeared in the regions of greatest energy, so the distortions in these regions were invisible. As Figure 3e shows, the visibility-weighted local SDSIM map had a better performance in predicting the locations and perceived visible intensities of distortions.

### 3.3. Appearance Distortion Detection Strategy

When an image is of low quality, visual masking is less important for image quality judgment; on the contrary, when the image distortion exceeds the threshold, the degree of quantification of the distortion to reduce the appearance of the image subject can better simulate the visual system perceived distortion. Therefore, in this type of distortion, the HVS’s judgment on the image was switched to a judgment based on the appearance of the image.

To model this appearance perception mechanism of the visual system, a method based on local statistics was developed, which used a multiscale log-Gabor filter response for statistical calculation. The use of this type of statistical model to capture the appearance of texture has been used in various image processing studies. In addition, existing research showed that log-Gabors better simulated and modeled simple cells in the primary visual cortex. In the processing of texture, the change in pixel-based statistics was less obvious than that in the log-Gabor filter response-based statistics.

#### 3.3.1. Log-Gabor Decomposition

Both original and halftone images are first transformed into a set of sub-bands using a log-Gabor filter bank. By calculating the inverse Discrete Fourier Transform (DFT) of the product of the image DFT and the following two-dimensional frequency response, filtering for obtaining sub-bands are performed in the frequency domain.

The original image and the halftone image are decomposed by log-Gabor by multiplication in the frequency domain. $\left\{{\overset{´}{c}}_{s,o}\right\}$ represents the set of log-Gabor sub-bands calculated for either the original or halftone image; here, each sub-band ${\overset{´}{c}}_{s,o}\in R_{M\times N}$ has the size of the images. The log-Gabor decomposition is calculated using five scales s=1,…,5, and 4 orientations o=1,…,4; thus, each image has 20 sub-bands. This decomposition is applied to both the filtered original image $I_{org}$ and the halftone image $I_{dst}$ to obtain the sub-band sets $\left\{{\overset{´}{c}}_{s,o}^{org}\right\}$ and $\left\{{\overset{´}{c}}_{s,o}^{dst}\right\}$.

#### 3.3.2. Compare Sub-Band Statistics

By comparing the local sub-band statistics of the original image with the corresponding local sub-band statistics of the distorted image, the local statistical difference map η(p) was computed. For each 16 × 16 block, the difference in standard deviation and skewness of the corresponding sub-band coefficients of the block were calculated as:

(21) $$\eta (p)=\sum_{s=1}^{5}\sum_{o=1}^{4}w_{s}\left[\right|\sigma_{s,o}^{org}\left(p\right)-\sigma_{s,o}^{dst}\left(p\right)|+|\varsigma_{s,o}^{org}\left(p\right)-\varsigma_{s,o}^{dst}\left(p\right)\left|\right]$$

where $\sigma_{s,o}\left(p\right)$ and $\varsigma_{s,o}\left(p\right)$ represent the standard deviation and skewness of the 16 × 16 sub-band coefficients corresponding to scale *s* and orientation *o*, respectively, and corresponded to block *p* at a certain location. The fixed scale weights $w_{s}=0.5,0.75,1,5,$ and 6 are used to explain that the HVS-preferred coarse scales rather than fine scales. The final scalar value of the perceived distortion is given as:

(22) $$d_{appearance}={\left[\frac{1}{P}\sum_{p}{\eta (p)}^{2}\right]}^{\frac{1}{2}}$$

where the summation is for all blocks, and *P* represents the total number of blocks. $d_{sdsim}=0$ meant no distortion perceived, and an increase in the $d_{sdsim}$ value indicates an increase in perceived distortion, which reduces visual quality.

### 3.4. Adaptive Combination of Two Strategies

The adaptive combination method is based on the study in [19] that the observer intends to interactively judge low-quality and high-quality images. Here, our proposed index uses a weighted geometric mean of $d_{sdsim}$ and $d_{appearance}$, given by:

(23) $$Index={\left(d_{sdsim}\right)}^{\alpha }{\left(d_{appearance}\right)}^{1-\alpha }$$

Here, α is computed via:

(24) $$\alpha =\frac{1}{1+\beta_{1}(d_{sdsim}{)}^{\beta_{2}}}$$

The parameters $\beta_{1}$ and $\beta_{2}$ were provided in [16]. For the database of A57, the optimization values of these parameters are $\beta_{1}=0.467$ and $\beta_{2}=0.130$.

## 4. Experiment

### 4.1. Subjective Image Database and Processing

**Figure 4:**
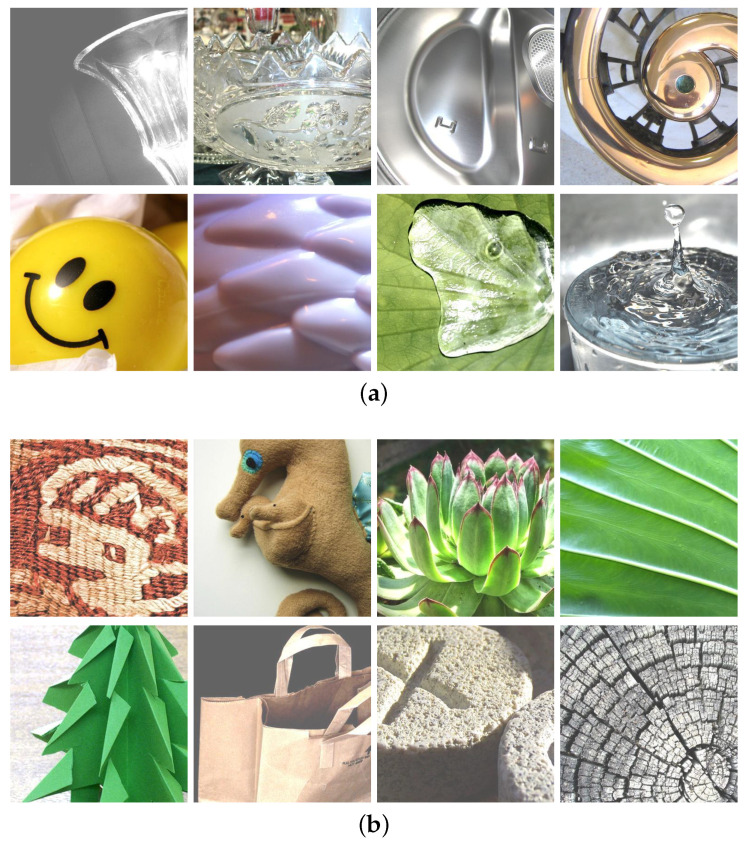
Test samples: (**a**) glossy images: glass, metal, plastic, and water droplets; (**b**) non-glossy images: fabric, plants, paper, stone, and wood.

The proposed algorithms were implemented using MATLAB (R2021a) running on the Mac OS. The experiments were conducted using 100 images from the Flickr Material Database [29]. The images we selected included not only pure glossy and non-glossy images but also images that contained both glossy and non-glossy parts, such as images containing textured leaves and water drops. Figure 4 shows the partial glossy and non-glossy images. Based on the above images, we conducted subjective and objective experiments, respectively. Then, we calculated the correlation between them. To verify the correlation between them, we ranked the observers’ scores from high to low, and the corresponding objective scores were automatically ranked. Then, we specified the 50 data pairs with the highest subjective score as the score of the non-glossy image and the other 50 data pairs as the score of gloss images.

**Figure 5:**
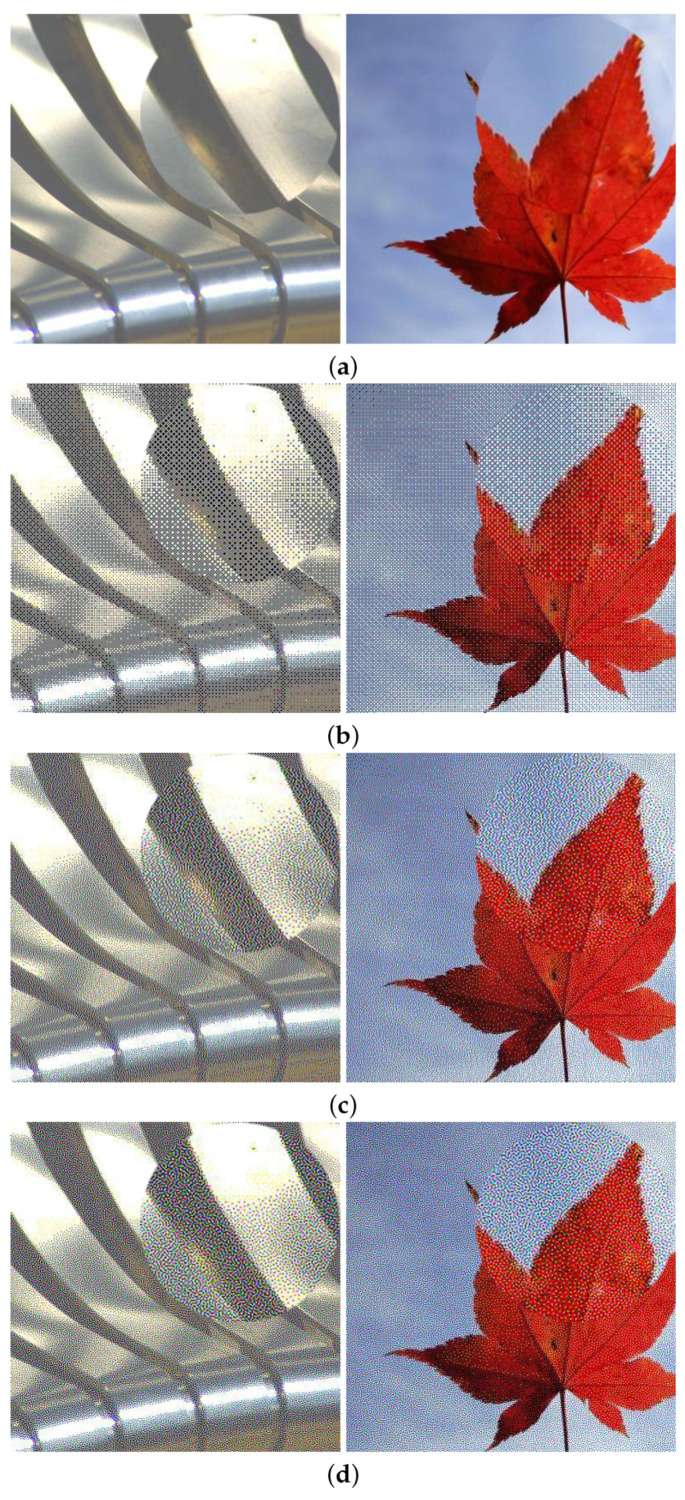
Examples of samples. Left and right images show gloss and non-gloss images, respectively: (**a**) original images; (**b**) halftone images by dithering; (**c**) halftone images by Floyd; (**d**) halftone images by DBS.

For comparison, we selected several halftone algorithms to process the selected 100 images. These algorithms included three different halftone algorithms: dithering [27], Floyd [1], and direct binary search (DBS) [30]. These different processing data were treated as three diverse types of distorted images. Different halftone technologies often produced different halftone effects. We process the color image by using color separation where an RGB image is divided into separate R, G, and B components with sRGB. After being processed by the halftone algorithm, the components are finally synthesized into an RGB image. In this process, the RGB color space is used. It was believed that the ED halftone reproduced more details of the original image than the dithering algorithm. Therefore, we believed that the distortion effect of the ED algorithm was less than that of the dithering halftone algorithm. Figure 5 shows an example of the application of each algorithm to the enlarged images. The size of the images is 384 × 384 pixels. The error diffusion and dithering halftone dots occupy 1 pixel and 4 × 4 pixels, respectively. In dither halftoning, the dot size determines the image quality of halftone and affects the printed image. Too large a dot size will cause the image to lose too many details and also affect the output effect of printer. Too small a dot size will appear as regular fence phenomenon, reducing the image quality.

In the actual printing process, various effects (e.g., dot gain) occur, depending on the characteristics of the paper. They can be perceived differently depending on lighting effects. Therefore, it is difficult to isolate how these effects impact the subjective evaluation of the printed material. Therefore, we evaluated the digital data presented on the display device. The original image and the halftone image were displayed simultaneously, and the observer scored them. Observers were asked to assign a score from 0 to 100 for each test image pair. The viewing distance was designed to be equivalent to the retinal image of a 600 dpi print at 24 cm. Five observers participated in a subjective observer experiment with normal vision.

For comparison, we chose several image quality evaluation algorithms for the experiments. We calculated the peak signal-to-noise ratio (PSNR), color structural similarity (CSIM) [21], MAD [16], and the proposed index to compare the correlation between the results of each index and the subjective observer score. We showed normalized index values for MAD and the proposed method because the indexes belonged to [0,∞].

### 4.2. Experimental Procedure

**Figure 6:**
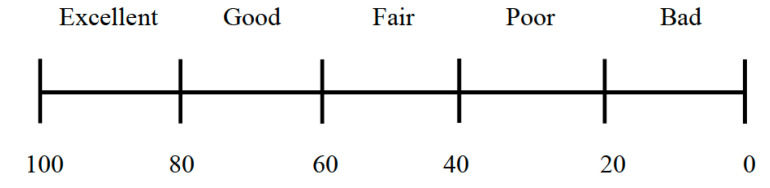
Rating scale used in subjective experiment.

There are five students (three males and two females; age 27.2 ± 6.65) who participated in the experiment. One student (the author) had significant experience with the subjective assessment of printing image quality. The other four students were all non-experts in printing and image quality assessment. All students were screened prior to participation for normal, or corrected to normal, visual acuity and normal color vision. Image pairs were presented on the display for a duration of 10s. A reference image and a halftone image are placed on the left and right, respectively. Observers were given instructions to judge the reproduction of objects’ glossiness in images and to provide corresponding scores. After observing an image pair, observers were asked to provide corresponding scores to the image pair using a continues horizontal scale, as depicted in Figure 6. The scores corresponding to the above interval are 100, 80, 60, 40, 20, and 0, respectively. Scores ranged from 100 to 0 using a continuous horizontal scale, with 0 representing poor reproduction and 100 representing objects that were most similar to the original image.

### 4.3. Results and Discussion

To assess the consistency by five observers measuring the same quality, we calculated the interclass correlation coefficient (ICC) for the inter-observer consistency. ICC is a descriptive statistic that can be used when quantitative measurements are made on units that are organized into groups. It describes how strongly units in the same group resemble each other. Before we calculated the consistency with the subjective score, we conducted a reliability study to evaluate the inter-observer test-retest reliability. Based on 100 images of each halftone, we repeated the subjective experiment for each image pair with five observers, we analyzed the data using a single-measurement, absolute-agreement, two-way mixed-effects model. For the different halftones, our ICC report is summarized in Table 1.

As shown in Table 1, we concluded that the test-retest reliability of our subjective experiment is “moderate” and “good”.

**Figure 7:**
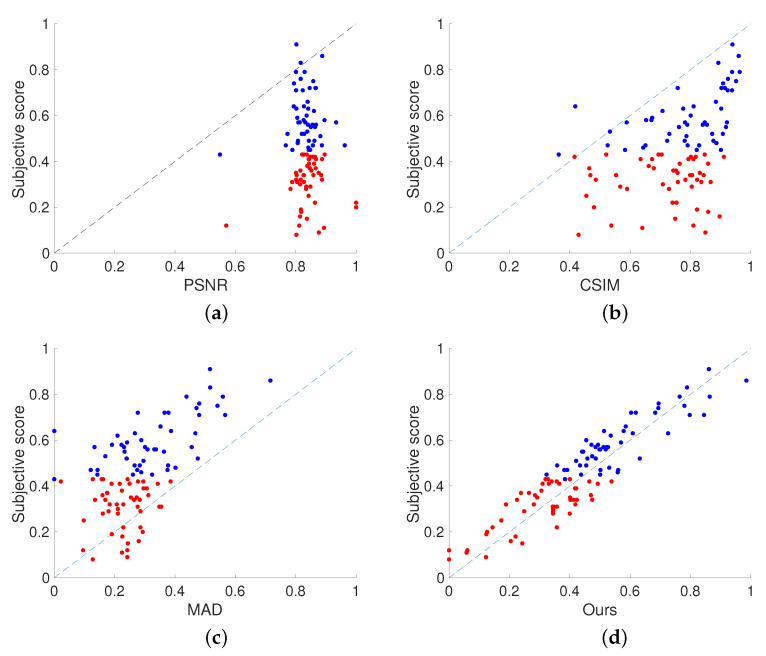
Scatter plots of the PSNR, CSIM, MAD, and proposed metric for test samples halftoned by Floyd: (**a**) PSNR, (**b**) CSIM, (**c**) MAD, and (**d**) Ours.

**Figure 8:**
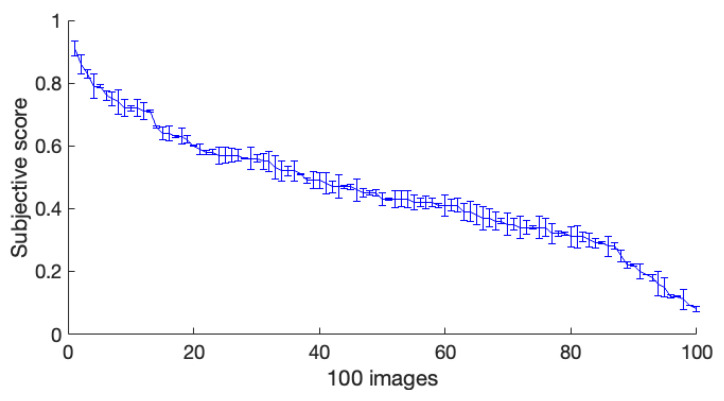
Subjective scores with error bars based on Floyd from 5 observers.

The Pearson correlation coefficient (PCC) was the most common measure of predictive performance. Here, we used PCC to calculate the correlation between the observer scores and objective index values. To distinguish the effects of metrics on glossy and non-glossy images, we sorted the subjective data in descending order, and the objective data were sorted accordingly. The first 50 images of the arranged data were classified as non-glossy images, and the rest were classified as glossy images. The former was represented by red points, the latter by blue points. Figure 7 shows the scatter plots of PSNR, CSIM, MAD, and the proposed image glossiness metric for 100 images based on Floyd. In all graphs, each point represented a test image. Figure 8 shows the error bar of the subjective score based on Floyd from the five observers. In Figure 7, the vertical axis represents the subjective ratings of the perceived distortions, and the horizontal axis represents the metrics. As Figure 7 shows, the proposed index evaluated the glossiness correctly compared to other indices. Comparable results were obtained for the other halftone methods.

Table 2, Table 3 and Table 4 show the PCC between four kinds of different metrics and subjective observer scores based on three different halftoning algorithms (dither, Floyd, and DBS). We separately calculated the correlation coefficients of glossy, non-glossy, and 100 images. Based on different distortion types, we separately calculated the PCC of glossy images, non-glossy images, and 100 images. The proposed index was consistent with the subjective evaluation regardless of the glossiness of the object.

## 5. Conclusions

We proposed an effective index that explicitly separated structure detection and appearance for the glossiness of objects in halftone images. For structure detection, the SDISM algorithm was developed, which worked effectively for structural distortion. Two important HVS characteristics, i.e., CSF and contrast masking, were incorporated into the metric to better simulate HVS responses to visual inputs. We proposed two simple quality measures, SDSIM and appearance perception, which were responsible for correlating structure, skewness, and standard deviation. Through experiments using 300 test images halftoned from 100 images in FMD, we demonstrated the effectiveness of the proposed index, which was consistent with the subjective evaluation scores regardless of the glossiness of the object.

We will continue to investigate effective indices for other appearances of objects, such as perceptual transparency. Our future research will consider more advanced color spaces, such as S-CIELAB and iCAM. New material perception models will also provide potential research possibilities, such as the new material perception model proposed in paper [31]. Furthermore, we will further verify the effectiveness of our index in the actual printing with different papers and lighting environments.

## Figures and Tables

**Table 1:** ICC and 95% confident interval for different halftone.

Halftone	ICC	95% Confident Interval
Dithering	0.78	0.72–0.84
Floyd	0.82	0.74–0.85
DBS	0.85	0.76–0.87

**Table 2:** Pearson correlation coefficient for PSNR, CSIM, MAD, and Ours based on dithering.

Index	Glossiness Images	Non-Glossiness Images	All 100 Images
PSNR	0.2275	0.2375	0.102
CSIM	0.1944	−0.1492	0.0038
MAD	0.2566	−0.0244	0.0355
Ours	0.7563	0.7707	0.9066

**Table 3:** Pearson correlation coefficient for PSNR, CSIM, MAD, and Ours based on Floyd.

Index	Glossiness Images	Non-Glossiness Images	All 100 Images
PSNR	0.1595	0.0626	0.0029
CSIM	0.0668	0.5067	0.4053
MAD	0.0896	0.6542	0.5788
Ours	0.7662	0.8716	0.9131

**Table 4:** Pearson correlation coefficient for PSNR, CSIM, MAD, and Ours based on DBS.

Index	Glossiness Images	Non-Glossiness Images	All 100 Images
PSNR	−0.0954	−0.2506	−0.2509
CSIM	0.6294	0.5453	0.7132
MAD	0.5735	0.6154	0.7045
Ours	0.7311	0.8881	0.9101

## Data Availability

Data are contained within the article.

## References

[1] Floyd R. (1975). An Adaptive Algorithm for Spatial Grey Scale. Soc. Inf. Display.

[2] Floyd R. (1976). An adaptive algorithm for spatial grey scale. Proc. Soc. Inf. Display.

[3] Damera-Venkata N., Evans B.L. (2001). Adaptive threshold modulation for error diffusion halftoning. IEEE Trans. Image Process..

[4] Monga V., Evans B.L. Tone dependent color error diffusion. Proceedings of the 2004 IEEE International Conference on Acoustics, Speech, and Signal Processing.

[5] Pang W.M., Qu Y., Wong T.T., Cohen-Or D., Heng P.A. Structure-aware halftoning. Proceedings of the ACM SIGGRAPH 2008 Special Interest Group on Computer Graphics and Interactive Techniques Conference.

[6] Akarun L., Yardunci Y., Cetin A.E. (1997). Adaptive methods for dithering color images. IEEE Trans. Image Process..

[7] Xia M., Hu W., Liu X., Wong T.T. Deep halftoning with reversible binary pattern. Proceedings of the IEEE/CVF International Conference on Computer Vision.

[8] Leloup F.B., Audenaert J., Hanselaer P. (2019). Development of an image-based gloss measurement instrument. J. Coatings Technol. Res..

[9] Anderson B.L., Kim J. (2009). Image statistics do not explain the perception of gloss and lightness. J. Vis..

[10] Ferwerda J.A., Pellacini F., Greenberg D.P. Psychophysically based model of surface gloss perception. Proceedings of the Human Vision and Electronic Imaging VI. International Society for Optics and Photonics.

[11] Thompson W., Fleming R., Creem-Regehr S., Stefanucci J.K. (2011). Visual Perception from a Computer Graphics Perspective.

[12] Toscani M., Valsecchi M., Gegenfurtner K.R. (2013). Optimal sampling of visual information for lightness judgments. Proc. Natl. Acad. Sci. USA.

[13] Wiebel C.B., Toscani M., Gegenfurtner K.R. (2015). Statistical correlates of perceived gloss in natural images. Vis. Res..

[14] Pont S.C., Koenderink J.J. (2005). Reflectance from locally glossy thoroughly pitted surfaces. Comput. Vis. Image Underst..

[15] Wang Z., Bovik A.C., Sheikh H.R., Simoncelli E.P. (2004). Image quality assessment: From error visibility to structural similarity. IEEE Trans. Image Process..

[16] Larson E.C., Chandler D.M. (2010). Most apparent distortion: Full-reference image quality assessment and the role of strategy. J. Electron. Imaging.

[17] Li S., Zhang F., Ma L., Ngan K.N. (2011). Image quality assessment by separately evaluating detail losses and additive impairments. IEEE Trans. Multimed..

[18] Ding K., Ma K., Wang S., Simoncelli E.P. (2020). Image quality assessment: Unifying structure and texture similarity. arXiv.

[19] Ulichney R. (1987). Digital Halftoning.

[20] Lau D.L., Arce G.R. (2018). Modern Digital Halftoning.

[21] Lee J., Horiuchi T. (2008). Image quality assessment for color halftone images based on color structural similarity. IEICE Trans. Fundam. Electron. Commun. Comput. Sci..

[22] Mannos J., Sakrison D. (1974). The effects of a visual fidelity criterion of the encoding of images. IEEE Trans. Inf. Theory.

[23] Winkler S. Visual quality assessment using a contrast gain control model. Proceedings of the 1999 IEEE Third Workshop on Multimedia Signal Processing (Cat. No. 99TH8451).

[24] Osberger W., Bergmann N., Maeder A. An automatic image quality assessment technique incorporating higher level perceptual factors. Proceedings of the 1998 International Conference on Image Processing ICIP98 (Cat. No. 98CB36269).

[25] Násánen R. (1984). Visibility of halftone dot textures. IEEE Trans. Syst. Man Cybern..

[26] Damera-Venkata N., Kite T.D., Geisler W.S., Evans B.L., Bovik A.C. (2000). Image quality assessment based on a degradation model. IEEE Trans. Image Process..

[27] Verevka O., Buchanan J.W. (1999). Halftoning with image-based dither screens. GI.

[28] Mullen K.T. (1985). The contrast sensitivity of human colour vision to red-green and blue-yellow chromatic gratings. J. Physiol..

[29] Sharan L., Liu C., Rosenholtz R., Adelson E.H. Flickr Material Database. https://people.csail.mit.edu/lavanya/fmd.html.

[30] Analoui M., Allebach J.P. Model-based halftoning using direct binary search. Proceedings of the SPIE/IS&T 1992 Symposium on Electronic Imaging: Science and Technology.

[31] Serrano A., Chen B., Wang C., Piovarči M., Seidel H.P., Didyk P., Myszkowski K. (2021). The effect of shape and illumination on material perception: Model and applications. ACM Trans. Graph. (TOG).

